# Characterization of *Haemonchus contortus* Excretory/Secretory Antigen (ES-15) and Its Modulatory Functions on Goat Immune Cells In Vitro

**DOI:** 10.3390/pathogens9030162

**Published:** 2020-02-27

**Authors:** Muhammad Ehsan, Javaid Ali Gadahi, Muhammad Waqqas Hasan, Muhammad Haseeb, Haider Ali, Ruofeng Yan, Lixin Xu, Xiaokai Song, Xing-Quan Zhu, Xiangrui Li

**Affiliations:** 1 MOE Joint International Research Laboratory of Animal Health and Food Safety, College of Veterinary Medicine, Nanjing Agricultural University, Nanjing 210095, China; mehsan124@gmail.com (M.E.); waqqas.hasan@gmail.com (M.W.H.); muhammadhaseeb73@gmail.com (M.H.); 2018207074@njau.edu.cn (H.A.); yanruofeng@njau.edu.cn (R.Y.); xulixin@njau.edu.cn (L.X.); songxiaokai@njau.edu.cn (X.S.); 2 State Key Laboratory of Veterinary Etiological Biology, Key Laboratory of Veterinary Parasitology of Gansu Province, Lanzhou Veterinary Research Institute, Chinese Academy of Agricultural Sciences, Lanzhou 730046, China; xingquanzhu1@hotmail.com; 3 Department of Veterinary Parasitology, Sindh Agriculture University, Tandojam, Sindh 70050, Pakistan; drgadahi@yahoo.com

**Keywords:** *Haemonchus contortus*, 15 kDa, goat, PBMCs, host–parasite interactions, immunomodulation, cytokines

## Abstract

Small size excretory/secretory (ES) antigens of the *Haemonchus contortus* parasite have intense interest among researchers for understanding the molecular basis of helminths immune regulation in term of control strategies. Immunomodulatory roles of *H. contortus* ES-15 kDa (HcES-15) on host immune cells during host–parasite interactions are unknown. In this study, the HcES-15 gene was cloned and expression of recombinant protein (rHcES-15) was induced by isopropyl-ß-d-thiogalactopyranoside (IPTG). Binding activity of rHcES-15 to goat peripheral blood mononuclear cells (PBMCs) was confirmed by immunofluorescence assay (IFA) and immunohistochemical analysis showed that *H. contortus* 15 kDa protein localized in the outer and inner structure of the adult worm, clearly indicated as the parasite’s ES antigen. The immunoregulatory role on cytokines production, cell proliferation, cell migration, nitric oxide (NO) production, apoptosis, and phagocytosis were observed by co-incubation of rHcES-15 with goat PBMCs. The results showed that cytokines IL-4, IL-10, IL-17, the production of nitric oxide (NO), PBMCs apoptosis, and monocytes phagocytosis were all elevated after cells incubated with rHcES-15 at differential protein concentrations. We also found that IFN-γ, TGF-β1, cells proliferation and migration were significantly suppressed with the interaction of rHcES-15 protein. Our findings indicated that low molecular ES antigens of *H. contortus* possessed discrete immunoregulatory roles, which will help to understand the mechanisms involved in immune evasion by the parasite during host–parasite interactions.

## 1. Introduction

Haemonchosis is an important disease of the trichostrongyle nematode parasite *Haemonchus contortus* and is liable for huge economic and productive losses due to its blood feeding behavior in the abomasum of domestic livestock [1]. The control of nematode parasites through anthelmintics and their resistance to these drugs, provide great interest among researchers to develop vaccines as an alternative source of prevention [2]. During the developmental stage of the parasite, especially *H. contortus* releases varieties of antigens into the host and these antigens are commonly called excretory/secretory proteins (ESPs) as important vaccine candidates due to their high immunogenic nature [3,4].

The identification of excretory/secretory (ES) antigens of some parasite families; like Trichostrongyloidea and Ancylostomatoidea by multiple identification tools [3,5], and their potential to mediate protective immune responses were studied [6]. *H. contortus* and its closely related nematode parasites for example; *Teladorsagia circumcincta* [7], *Ostertagia ostertagi* [8], and *Cooperia species* [9] release large number of ES products, which are assumed to have complex functional activity.

Due to the emergence of drug resistance, many researchers have been focusing on ES molecules as protective antigens from the gut of the parasite or so called ‘hidden antigens’ and their immunogenic nature serve as an alternative source of protection against helminths infection and/or disease [10,11,12,13,14]. In previous studies, two low molecular weight antigens from *H. contortus*, ES-15, and its closely related ES-24 were purified from ES products and determined for their protective potential against parasite infection in vivo [1,15]. Previously, the number of Hc-15 isoforms were identified from *H. contortus* ESPs (HcESPs) by hyperimmune sera and assumed to have a possible precise role during pathogenesis and/or in the immune evasion mechanism of the parasite [3]. Furthermore, it was demonstrated that vaccination with low molecular weight ES antigens induced Th2 type immune responses [16,17] and hypersensitivity reactions [18]. However, their effects on host immune cells are still unknown.

Previously, we identified that HcESPs showed a decreased immune functional role on goat peripheral blood mononuclear cells (PBMCs) in vitro with suppression of IL-4, IFN-γ, nitric oxide production, and elevated level of IL-10 cytokine, inflammatory cytokine IL-17, and migration percentage [19]. In our recent study, low molecular weight *H. contortus* recombinant ES protein 24 (rHcES-24) modulated the immune functions of goat PBMCs by increased production of IL-4, IL-10, IL-17 cytokines, cell migration, and suppressed level of IFN-γ, PBMCs proliferation, and NO production in vitro [20].

In the present study, *H. contortus* ES antigen (rHcES-15) was cloned, characterized, and its immuno-regulatory roles on goat PBMCs were highlighted, which could provide new insight to understand biology and the immunological functional role of this antigen, during host–parasite interactions.

## 2. Results

### 2.1. Cloning of HcES-15 Gene

The amplified fragments of the HcES-15 gene were successfully obtained by PCR from *H. contortus* cDNA with specific pair of primers, and a fragment of the correct size of 414 bp was obtained. The PCR amplicons were purified and successfully ligated into the pMD19-T cloning vector which was confirmed by restriction enzyme digestion with *BamH* I and *EcoR* I restriction site enzymes (Supplementary File: Figure S1).

### 2.2. Construction and Identification of the Recombinant pET32a (+)-HcES-15 Plasmid

The correct fragment of HcES-15 after sequencing was then inserted into *BamH* I/*EcoR* I sites of the pET32a (+) vector. The recombinant pET32a(+)-HcES-15 plasmid produced a fragment of about 414 bp with restriction enzyme digestion, which is equal to the molecular mass of HcES-15 (Supplementary File: Figure S1). These results indicated that HcES-15 was successfully inserted into the pET32a vector.

### 2.3. Sequence and Phylogenetic Analysis of HcES-15

The isolated sequences were confirmed as the HcES-15 gene by BLASTx and the translated protein sequence by BLASTp encodes 128 amino acids residues. The BLAST system indicated that query sequence had high identity only with the *H. contortus* ES protein and less extent with the *Trichostrongylus colubriformis* glycoprotein (Table 1). Multiple sequence alignment and the phylogenetic tree of the deduced protein sequence of HcES-15 with available sequences on NCBI database (Supplementary File: Figure S2), indicated that HcES-15 was highly related to *H. contortus* 15 kDa ES protein (100%, 92%, 89%, 85%, and 81%), and *H. contortus* protein 15 (p15), 97%, 92%, 88%, and 83%. Whereas, very low similarity was found with the *Ostertagia ostertagi* putative L3 ES protein and *T. colubriformis* 30 kDa antigenic glycoprotein, 26% and 38% respectively. The amino acid analysis using the SignalP program revealed the presence of an obvious signal peptide which possessed a cleavage site between position 19 and 20 (Supplementary File: Figure S3). Whereas, no transmembrane domain was found in the deduced protein (Supplementary File: Figure S4).

### 2.4. Expression and Purification of rHcES-15 Protein

**Figure 1:**
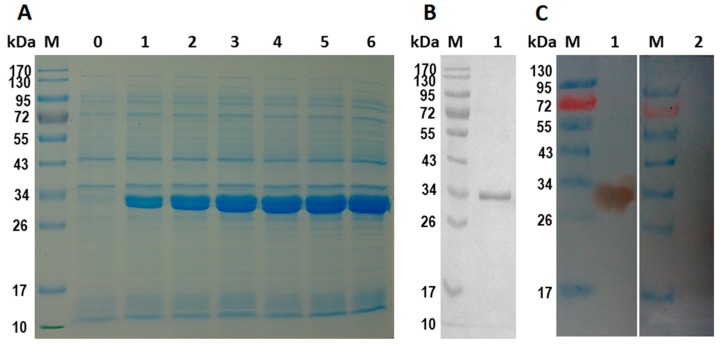
Expression, purification, and immuno blot analysis of recombinant HcES-15. M: standard molecular weight protein marker, (**A**) Lane 0: recombinant expression vector before isopropyl-ß-d-thiogalactopyranoside (IPTG) induction. Lane 1-6: protein expression after IPTG induction at different time points. (**B**) Lane 1: purified rHcES-15 protein. (**C**) Lane 1: Western blot analysis for purified rHcES-15 probed with rat anti- rHcES-15 sera. Lane 2: rHcES-15 probed with normal rat sera as control.

The isopropyl-ß-d-thiogalactopyranoside (IPTG) induced protein product of rHcES-15 expressed in *Escherichia coli* (*E. coli*) (BL21) cells showed a His tagged fusion protein on SDS-PAGE after Coomassie brilliant blue staining (Figure 1A) that was purified by chromatography on Ni-NTA and detected a single band with 33 kDa rather than calculated molecular mass of 15 kDa (Figure 1B). The expressed product was larger in size because of the fused 18 kDa vector protein of pET32a. By subtracting the size of the fused protein, the recombinant protein molecular weight was consistent with the deduced size of 15 kDa.

### 2.5. Western-Blot Analysis of rHcES-15 Protein

The results of the Western blot indicated that the recombinant HcES-15 protein was recognized by the immune sera, rat anti-rHcES-15 as a band of about 33 kDa but could not be recognized by the sera from normal rat (Figure 1C).

### 2.6. Binding of rHcES-15 to Goat PBMCs

**Figure 2:**
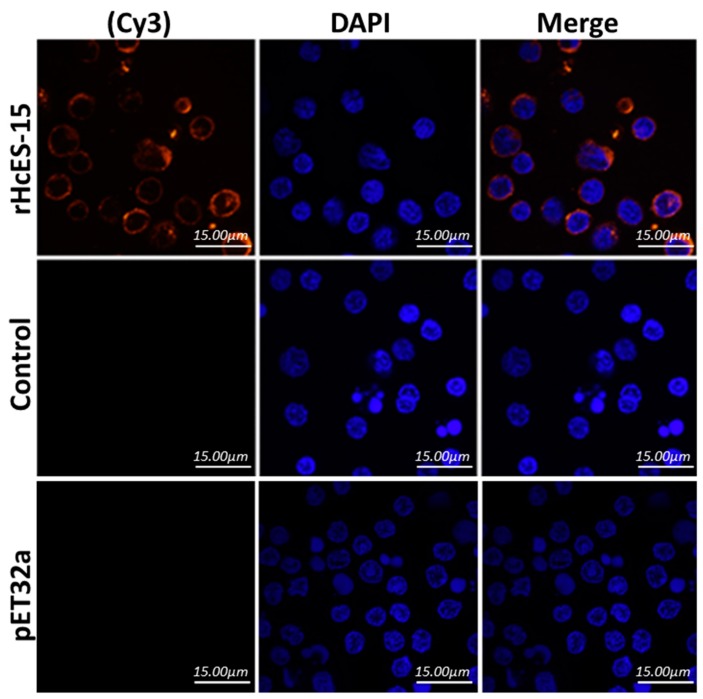
Binding confirmation of recombinant protein rHcES-15. The nuclei of the corresponding cells were visualized by DAPI (blue) staining, target proteins (red) were visualized by Cy3-conjugated secondary antibody and merge combination of red and blue channels. No protein binding was observed in control group. Scale bar 15 µm.

The binding of rHcES-15 with goat PBMCs was confirmed by using an immunofluorescence assay (IFA). As depicted in Figure 2, nuclei of the cells were shown with DAPI (blue fluorescence), and subsequently binding of target protein (rHcES-15) with Cy3 (red fluorescence) by confocal microscopy. The results revealed that rHcES-15 protein could bind on the surface of the cells to perform a specific immune functional role during host–parasite interaction. In the control group, no red fluorescence was observed (Figure 2).

### 2.7. Expression of HcES-15 in Adult Worms of H. contortus

**Figure 3:**
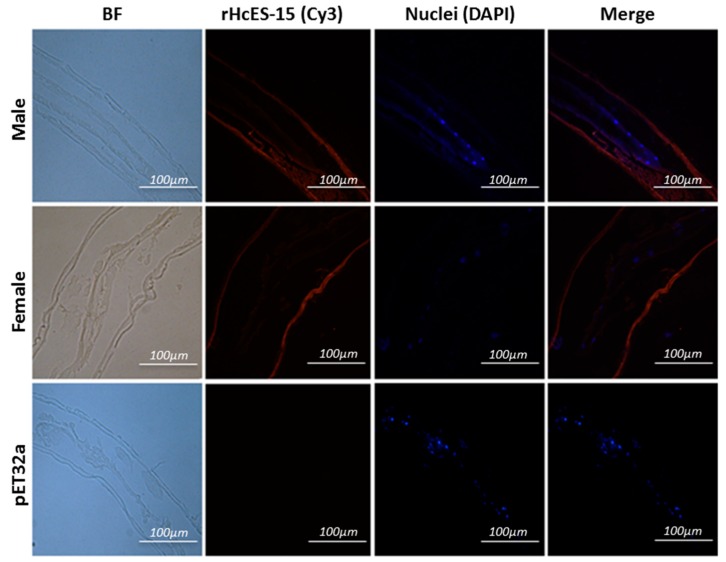
Expression of HcES-15 protein in the adult *H. contortus* by immunohistochemical analysis. The BF represents bright florescence. Target protein localization in male and female worms was detected by indirect immunofluorescence method using Cy3-conjugated secondary antibody. Nuclei were stained with DAPI (blue) and Merge combined DAPI and Cy3. No florescence was observed in control. Scale bar 100 µm.

The partial body longitudinal sections of the adult *H. contortus* male and female worm were used in an immunohistochemical assay to detect localization of HcES-15 (Figure 3). Various blue spots inside the body of worms clearly indicated the position of nuclei along the gut structure in both sections whereas, red florescence in the peripheral membrane structure and in the gut region of the parasite indicated HcES-15 as an ES antigen of *H. contortus*. No protein labeling was found in the control group.

### 2.8. Detection of the Cytokine Levels by ELISA

**Figure 4:**
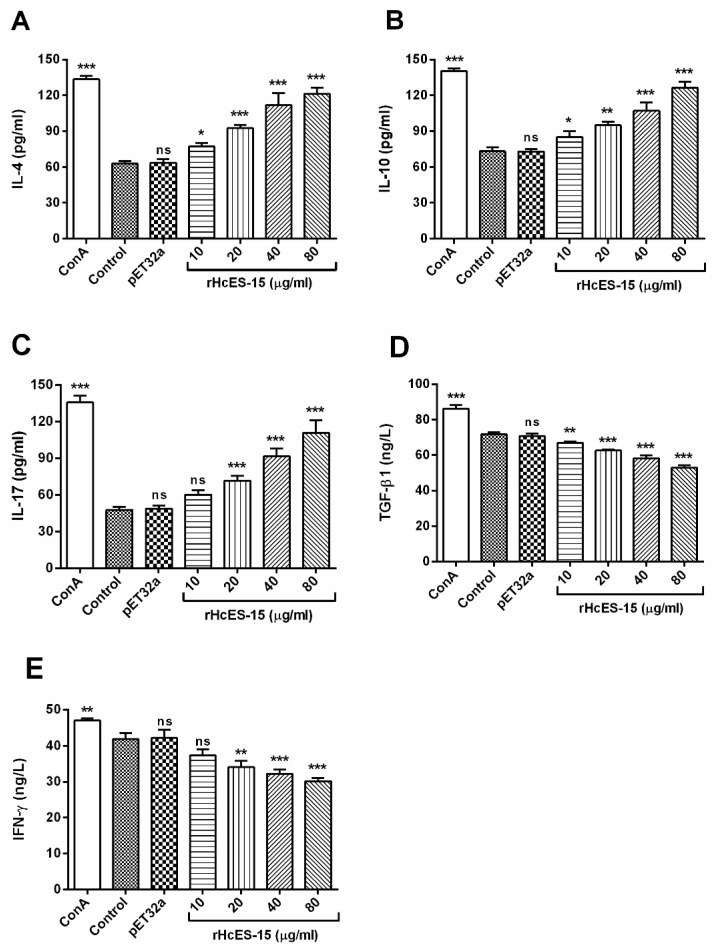
Analysis of the cytokines expression level by peripheral blood mononuclear cells (PBMCs) in vitro. The treatment group was stimulated with ConA (10 μg/mL) along with various concentrations of rHcES-15 and the control group was incubated with PBS (control) or ConA in presence of pET32a protein. Cytokine secretions for IL-4 (**A**), IL-10 (**B**), IL-17 (**C**), TGF-β1 (**D**) and IFN-γ (**E**) in the supernatant of cell cultures were quantified by ELISA. PBMCs used for all replicates of distinct treatments in each experimental repetition were derived from the same goat. The individual experiment was performed in triplicate (* *p* < 0.05, ** *p* < 0.01, and *** *p* < 0.001 versus the control), ns: no significant difference.

The effects of the rHcES-15 on the cytokines production by goat PBMCs were analyzed and results showed that the productions of IL-4 and IL-10 were increased significantly at dose dependent manners (*p* < 0.001) to that of control groups. The IL-17 secretion was also induced at 20, 40, and 80 µg/mL (*p* < 0.001) protein concentration but not significantly at 10 µg/mL (*p* > 0.05). Furthermore, the IFN-γ cytokines secretions with 20, 40, and 80 µg/mL of rHcES-15 decreased significantly (*p* < 0.001) whereas, 10 µg/mL concentration showed no effect (*p* > 0.05). However, effect of rHcES-15 significantly downregulated TGF-β1 production at dose dependent manner (*p* < 0.001) when compared to the PBS control group and pET32a group (Figure 4).

### 2.9. rHcES-15 Effected the Proliferation of Goat PBMCs

**Figure 5:**
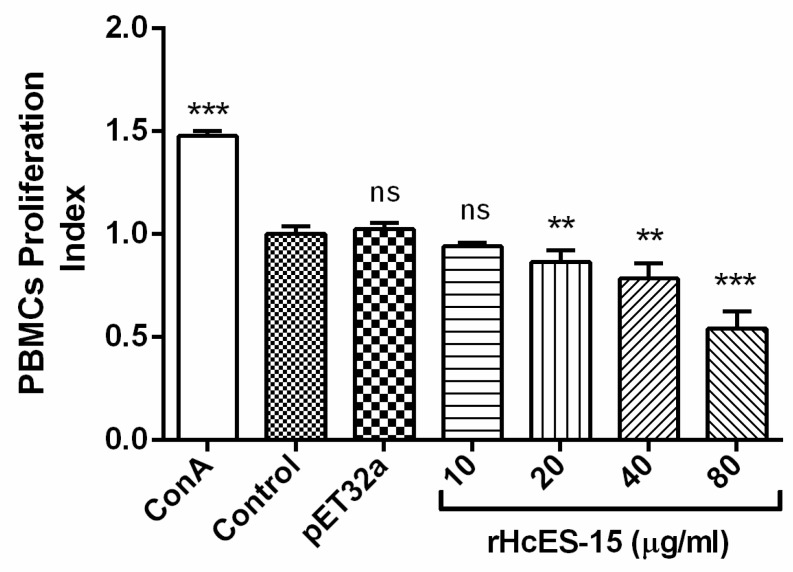
rHcES-15 effects the proliferation on goat PBMCs. The proliferation was measured by CCK-8 incorporation after stimulation of cells with ConA or along with serial concentrations of rHcES-15 at 37 °C and 5% CO_2_ for 72 h. The control group was incubated with PBS only or pET32a protein with ConA. The cell proliferation index was calculated considering the OD_450_ values in controls as 100%. PBMCs used for all replicates of distinct treatments in each experimental repetition were derived from the same goat. The data were analyzed from 3 independent experiments (** *p* < 0.01 and *** *p* < 0.001), ns: no significant difference.

Influence of rHcES-15 on cell proliferation was evaluated by cell counting kit 8 (CCK8) using a spectrophotometer. Results highlighted that there was no significant difference (*p* > 0.05) between the group treated with 10 μg/mL rHcES-15, pET32a group and PBS control group. The proliferation of PBMCs incubated with 20, 40, and 80 μg/mL of rHcES-15 protein concentrations was significantly suppressed (*p* < 0.001) as compared to control groups (Figure 5).

### 2.10. Cell Migration Assay

**Figure 6:**
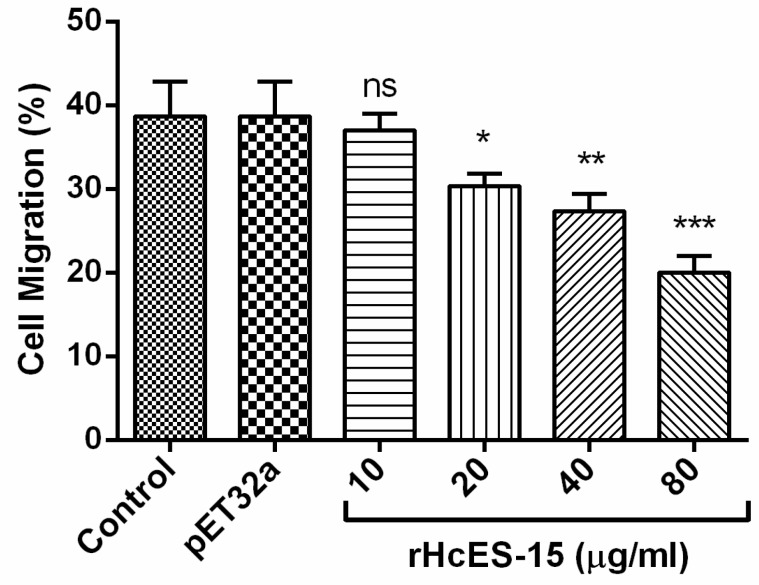
Effects of rHcES-15 on PBMCs migration. Cells were incubated with different concentrations of rHcES-15 as the treatment group or control buffer (PBS) and recombinant pET32a empty protein as control groups. Then, the random migration was determined. Statistical difference between the mean values was calculated using ANOVA. PBMCs used for all replicates of distinct treatments in each experimental repetition were derived from the same goat. The individual experiment was done in triplicate; * *p* < 0.05 and ** *p* < 0.01, *** *p* < 0.001 versus control.

PBMCs migration was evaluated by using Millicell^®^ insert (Corning, USA). The results showed that the percentage of migrated cells across the membrane in response to the rHcES-15 was significantly decreased at 20 µg/mL (30.33 ± 0.8819), 40 µg/mL (27.33 ± 1.202), and 80 µg/mL (20.00 ± 1.155) protein concentrations (*p* < 0.001) to that of PBS control and vector protein group, whereas not significantly in the group treated with 10 µg/mL (37.00 ± 1.155) protein concentration (*p* > 0.05) (Figure 6).

### 2.11. Nitric Oxide Production Assay

**Figure 7:**
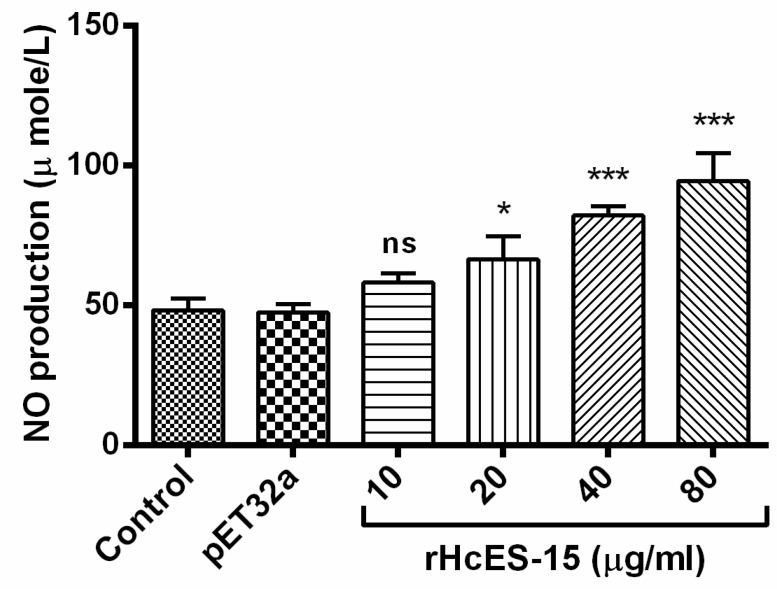
Impact of rHcES-15 on nitric oxide production by PBMCs in vitro. Cells were incubated with PBS, pET32a protein, and different concentrations of rHcES-15 for 24 h at 37 °C and 5% CO_2_. The nitrite concentration in the PBMCs was measured by using the Griess assay. PBMC used for all replicates of distinct treatments in each experimental repetition were derived from the same goat. The data were representative of three independent experiments (* *p* < 0.05, *** *p* < 0.001), ns: no significant difference.

Nitric oxide (NO) production by PBMCs treated with different concentrations of rHcES-15 was measured by using the total nitric oxide assay kit. Results showed that the rHcES-15 with 20, 40, and 80 µg/mL protein concentrations produced a significant level of NO in PBMCs (*p* < 0.001), whereas 10 µg/mL produced no significant production (*p* > 0.05) to that of the pET32a group and PBS control group (Figure 7).

### 2.12. rHcES-15 Protein Enhance Apoptosis of Goat PBMCs

**Figure 8:**
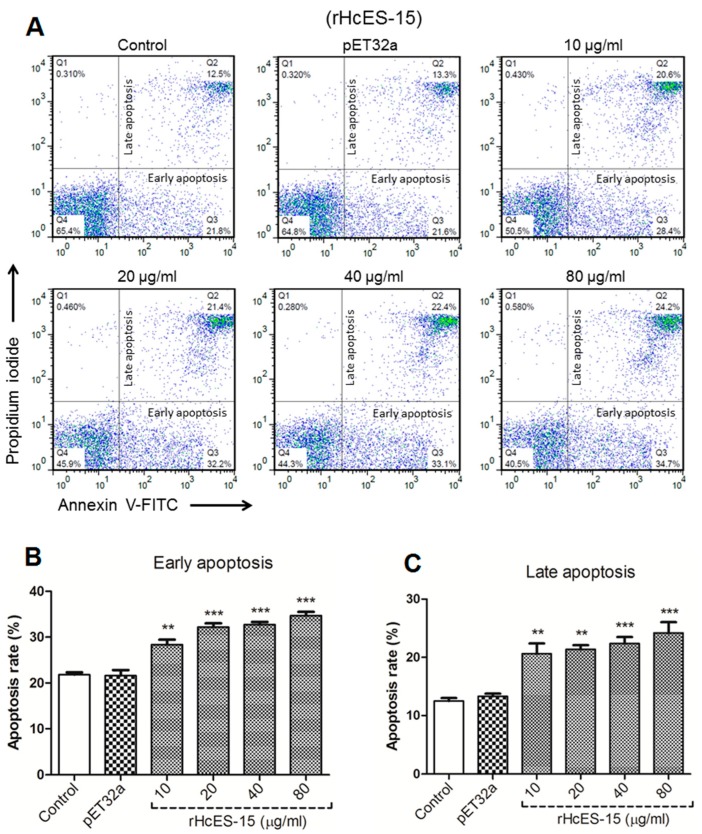
The flow cytometry analysis for PBMCs apoptosis. Apoptosis of PBMCs was determined by staining with annexin V and PI followed by flow cytometry. (**A**) The percentages of cells with different staining patterns are shown here. The results presented here are representative of three independent experiments. (**B**,**C**) Cells were incubated with different protein concentration to check its effect on early and late stage apoptotic percentage, and apoptosis was measured on four separate occasions. PBMCs used for all replicates of distinct treatments in each experimental repetition were derived from the same goat. Data are presented as the mean ± SEM (n = 3); an asterisk indicates treatment groups differ significantly (** *p* < 0.01, *** *p* < 0.001) to that of the control group.

The PBMCs were incubated with rHcES-15 protein with varying concentrations (10, 20, 40, 80 μg/mL) for 24 h and cells were stained with PI to detect apoptotic cells. The results showed that there was no significant change in annexin V positive pET32a empty protein group and PBS control groups (*p* > 0.05), whereas, rHcES-15 protein induced significantly in early as well as late stage apoptosis (*p* < 0.001) of the PBMCs at dose-dependent manner as compared to the control groups (Figure 8). Further evidence of flow cytometry analysis showed that spontaneous apoptosis exposure to different rHcES-15 protein levels was 49% at 10 μg/mL, 53.6% at 20 μg/mL, 55.5% at 40 μg/mL, and 58.9% at 80 μg/mL in goat PBMCs to that of the control and pET32a group which were 34.3% and 34.9% respectively, for a period of 24 h (Figure 8).

### 2.13. rHcES-15 Stimulated Phagocytosis of Goat Monocytes

**Figure 9:**
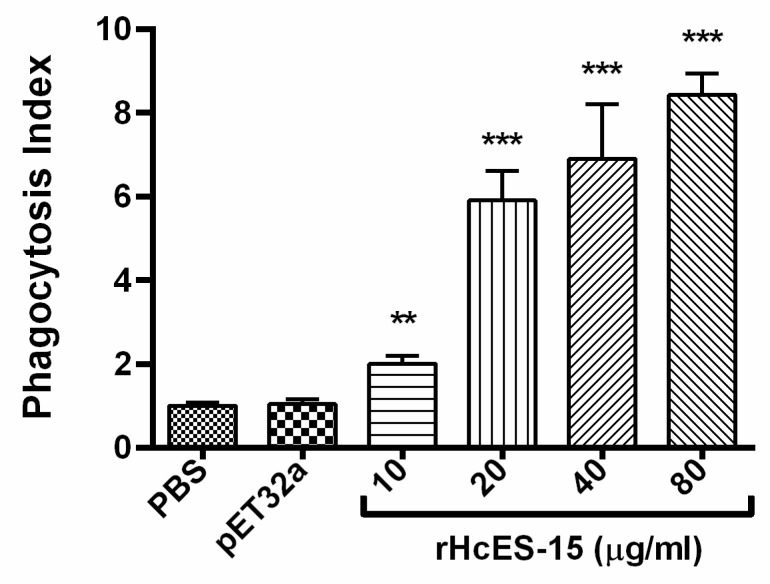
rHcES-15 increased Phagocytosis of goat PBMCs. Phagocytosis index was calculated considering the OD_540_ values in the control (PBS) group as 100%. The results are representative of triplicate experiments. PBMCs used for all replicates of distinct treatments in each experimental repetition were derived from the same goat. Data are presented as the mean ± SD (n = 3); an asterisk indicates treatment groups differ significantly (** *p* < 0.01, *** *p* < 0.001) compared to control groups.

The cell phagocytosis assay was performed to explore the effects of rHcES-15 on monocyte phagocytosis in goat PBMCs by up-taking FITC-dextran. Our results revealed that rHcES-15 protein showed a significantly high stimulatory effect on the monocytes phagocytosis at 10, 20, 40, and 80 μg/mL protein concentrations (*p* < 0.001). However, there was no significant difference induced by cells between the control group and pET32a treatment group (*p* > 0.05) (Figure 9).

## 3. Discussion

*H. contortus*, similar to other helminth parasites, actively exports or diffuses a variety of ES products into the host environment, which can challenge the host immune system through modulation or suppression of its functions [21]. In our previous proteomic study, numbers of HcESPs were identified and their interactions with host PBMCs were analyzed in vivo at different developmental stages of *H. contortus*. These interacting proteins were highly developmentally stage-specific proteins and their interactions with host cells resulted in complex regulation of the host immune cells [22]. Among these ESPs, the low molecular weight ES antigens (15 kDa) considered to induce a significant immune protection against *H. contortus* infection [15]. However, the precise roles of these small size proteins in immunobiology of the host–parasite interface still are unclear. In this study, gene encoding ES-15kDa from *H. contortus* was cloned, expressed, and its partial immune functional analysis was studied for the first time. Previously, Schallig and colleagues demonstrated that sheep with repeated *H. contortus* infection recognized 15 kDa ES molecules in crude and ES product of adult worms [23,24], which were detected by sera from hyper-immunized or challenged sheep [1]. In this study, *H. contortus* ES antigen, translated a recombinant protein with detected molecular mass of about 15 kDa, fused with the vector protein of 18 kDa on SDS-PAGE and was recognized by sera from rats experimentally infected with rHcES-15, suggesting that the recombinant protein contained appropriate antigenic determinants. We also determined sequence analyses for HcES-15, and did not find much similarity with known sequences from other parasites except *H. contortus*, but some matches with 30 kDa glycoprotein [25]. Our findings indicated that rHcES-15 is a novel protein with discrete potential against parasitic infection, and immunogenic properties in its structure need to be further researched.

During the host–parasite relationship, the immune system and its associated effector cells (Th1 and Th2), are mainly regulated by some cytokines that play important roles against nematodes infections [26,27]. It was considered that the main protective immune responses (Type 2) against helminthes including *H. contortus* are associated with secretion of IL-4 [28]. The pro-inflammatory cytokine, interferon gamma (IFN-γ) produced by Th1 cells was associated with regulation of cellular immunity against infection and participated in differentiation of Th1 and Th2 cells. The balanced state between Th1 and Th2 immune responses mediated by their representative cytokines IFN-γ and IL-4 respectively, could determine the immunity and pathogenesis during parasitic infection [29]. In our previous studies, we found that low molecular weight rHcES-24 and rHcARF1 increased the IL-4 secretion and decreased the production of IFN-γ in goat PBMCs [20,30]. Similarly, during evaluation of cytokines productions in response to HcESPs, IFN-γ secretion was downregulated [19]. In this study, the IFN-γ production was also decreased in goat PBMCs in response to the rHcES-15 protein. Thus, we can say that this protein might play a vital role in suppressive effects of ESPs on IFN-γ production. The T regulatory cells (Treg) and their associated cytokine IL-10 usually played suppressive functions on development of Th2 immune responses [31,32]. IL-10 cytokine, necessary for host defense, suppresses the innate and adaptive immune responses and limits the tissue damage caused by inflammation [33]. In accordance with previous studies on rHcES-24 and rHcARF1 [20,30], in this research, we demonstrated that rHcES-15 suppressed IFN-γ production, while a balanced Th1 and Th2 environment during host–parasite interface on increased IL-4, and IL-10 cytokine secretions in goat PBMCs are still subject to debate. Pro-inflammatory cytokine IL-17 produced by Th17 cells, is functionally associated with pathogenesis of various helminths parasites, and characterized as a tissue inflammatory modulator [34]. In our recent studies, IL-17 secretion either collectively, in the case of HcESPs, or individually (rHcES-24, rHcARF1 and rHcftt-2) was significantly increased in goat PBMCs in vitro [19,20,30,35]. In this investigation, a significantly increased level of Th17 secretion was detected in goat PBMCs in response to rHcES-15 protein. However, the induced Th17 cells differentiation and pathogenesis, might contribute to facilitate worm infection and needs to be further researched.

TGF-β is a multifunctional cytokine that controls proliferation and potentially regulates different immuno-modulatory activities, cellular differentiation, proinflammatory responses, and regulation of cell growth [36,37]. It was demonstrated that regulatory mechanism of cytokines, such as IL-10 and TGF-β, altered by host genetics, parasite developmental stage, and level of infection [32]. Moreover, it was suggested that TGF-β also played a dynamic role in inhibition of cell multiplication and stimulation of programmed cell death of numerous immune cell subsets [38]. Previously, a study showed that TGF-β also regulates Th17-cell differentiation and its related cytokine IL-17, both directly and indirectly by inhibiting T-cell differentiation [39], and it was also noted that Th17 cells can also be induced in cell culture without presence of TGF-β [40]. In the present investigation, the TGF-β1 level was decreased in goat PBMCs in response to rHcES-15 at a dose dependent manner. This multifaceted role of TGF-β might be driven by host genetic modification or the parasitic stage during complex host–parasite interaction, and is worth further investigation.

It was demonstrated that helminths could actively promote immune cell trafficking to the site of infection to initiate tissue damage, which could lead a favorable condition for worm survival [41]. Previously, two ES proteins, rHcES-24 and rHcfft-2, were found to increase mobility of PBMCs in vitro [20,35]. In contrary to previous work, in this study, decreased capacity of PBMCs migration induced by low molecular weight antigen (rHcES-15) suggested a mechanism by which this protein contributed to the worms evading host immunity, and needs to be further studied. NO has been suggested to be involved in the majority of parasitic infections including *H. contortus*, by mediating host non-specific defense through killing effect or retard parasitic growth [42]. Previously, it was reported that endogenous cytokine IL-17 was consistently involved in inducible nitric oxide synthase-mediated NO production [43]. In our previous study, the NO level was upregulated by the influence of rHcARF1 on goat PBMCs [30]. Similarly, another recent study supported our results in which rHc-GDC, an important constituent of HcESPs, increased NO production in goat PBMCs [44]. Consistent with previous studies, PBMCs incubated with rHcES-15 significantly increased NO production at maximum concentration. Our results indicated that increased level of NO in goat PBMCs might be associated with the up-regulation of IL-17, which might promote the Th17/NO-based inflammatory response and pathogenesis during *H. contortus* infection. Previous studies reported that ES antigens from helminths have divergent functions, particularly on reduced multiplication of host immune cells via direct cytolysis [45,46] or by promoting apoptosis [47,48]. A similar study also supported this evidence, that PBMCs incubated with live *Brugia malayi* L3 decreased total cell numbers by apoptosis [49]. In our recent studies, *H. contortus* ES products and purified ES molecules (rHcES-24, rHcftt-2) had a suppressive activity on PBMCs proliferation [19,20,35]. Consistently, in this study, we observed that high concentration of *H. contortus* ES-15 (40 and 80 µg/mL) increased the percentage of cell death, which in turn, decreased the viability of cells after 72 h of incubation. Phagocytosis is a host defense strategy in the immune system that contributes in clearance of apoptotic or pathogenic microorganisms, and it was shown that, numbers of galectin family members were involved in phagocytosis [50]. In the present study, effect of rHcES-15 on host cell phagocytic activity was evaluated, and found that phagocytosis of goat monocytes was significantly increased compared to the control group. However, the real mechanisms contributing to host cells apoptosis and phagocytosis associated with host immune responses and receptors involved in this process are worthy for further studies.

## 4. Materials and Methods

### 4.1. Ethics Statement

Animal experiments were conducted following the guidelines of the Animal Ethics Committee, Nanjing Agricultural University, China. All experimental protocols were approved by the Science and Technology Agency of Jiangsu Province. The approval ID is SYXK (SU) 2010-0005.

### 4.2. Animals and Parasites

The native crossbred goats 3 to 6 months old, from the research and teaching flock at Nanjing Agricultural University, were housed indoors with availability of microbes free feed and water ad libitum. All goats were dewormed twice at 2 weeks interval, to eliminate naturally acquired helminths infections. After two weeks, the standard parasitological techniques were applied to examine the helminths eggs in fecal samples of each goat microscopically, and goats showing no eggs were used in this study with maintained health conditions throughout the experiment. Adult worms used in the subsequent study, were collected from the infected donor goats as stated previously [51]. Three biological replicates (three goats), each consisting of three technical replicates (three replicates for each goat), were run for immune and functional studies including immunofluorescence assays, cytokine production, cell proliferation, nitric oxide production, migration assay, apoptosis, as well as phagocytosis activity.

Sprague Dawley (SD) rats (body weight ~150 g) were purchased from the Experimental Animal Center of Jiangsu, PR China (Qualified Certificate: SCXK 2008-0004) and were raised in a sterilized room and provided with sterilized food and water.

### 4.3. Isolation of PBMCs and Monocytes

PBMCs were separated from heparinized peripheral venous blood samples of dewormed healthy goats, by standard Ficoll-Hypaque (GE Healthcare, Munich, ND, USA) gradient centrifugation method [52] and washed twice in PBS (Ca^2+^/Mg^2+^-free, pH 7.4). The PBMCs were cultured in 24-well and 6-well (for monocytes) flat-bottomed culture plates (Corning, USA), containing cell culture medium Roswell Park Memorial Institute 1640 (RPMI 1640; GIBCO, Grand Island, NY, USA), supplemented with 10% heat inactivated fetal bovine serum (FBS), 100 U/mL penicillin, and 100 mg/mL streptomycin (GIBCO, USA), at 37 °C in 5% CO_2_ for 2 h. To collect monocytes, the non-adherent cells were aspirated by washing twice with PBS. The adherent cells were collected, adjusted to a density of 1 × 10^6^ cells/mL and trypan blue exclusion test was conducted for cell viability consistently >95% in all the experiments.

### 4.4. Synthesis of H. contortus cDNA

The total RNA isolation was carried out, followed by cDNA synthesis from adult worms of *H. contortus*, collected from the abomasum of donor goats. The worms were minced in a pre-chilled pestle and mortar with the addition of 1 mL of Trizol (Invitrogen) and homogenized for 30 min. Then, 200 µL of trichloromethane was added and the mixture was centrifuged at 10,000× *g* for 15 min at 4 °C. The RNA was precipitated from the supernatant by the addition of 0.25 volumes of isopropyl alcohol for each milliliter of Trizol and incubated at −20 °C for 30 min. The RNA was pelleted at 10,000× *g* at 4 °C for 10 min. The RNA pellets were dried after washing with 70% ethanol, resuspended in diethyl pyrocarbonate (DEPC) treated water which was used immediately for subsequent cDNA preparation. The cDNA was synthesized using the cDNA Kit (Takara Biotechnology, Kusatsu, Shiga, Japan) by reverse transcription reaction, according to the manufacturer’s instructions.

### 4.5. Molecular Cloning of HcES-15 Gene and Expression of rHcES-15 Protein

The complete open reading frame (ORF) of gene encoding for HcES-15 was amplified by reverse transcription polymerase chain reaction (RT-PCR) using a pair of primers that were designed from *H. contortus* 15 kDa ES protein mRNA, as shown in Table 2 with the following restriction enzyme-anchored (Italic) and protective bases-anchored primers: (Forward primer: 5′- AAA*GGATCC*ATGTTCTTCGCTTTTGC -3′ and Reverse primer: 5′- CTG*GAATTC*TCAGTTGGGGGTATTGT -3′). The PCR amplification was carried out using Thermocycler PCR Machine (Biometra, Dublin, Ireland) with the total reaction volume of 50 µL, containing 2 µL cDNA, 1.0 U Taq DNA polymerase (Takara Biotechnology, Dalian, China), 3.0 mM MgCl_2_, 400 µM dNTP mixture, 50 µM 10x LA PCR Buffer (Mg^2+^-Free), and 400 nM of each primer. The cyclic conditions were: initial denaturation at 94 °C for 5 min (1 cycle), denaturing at 94 °C for 1 min, annealing at 55 °C for 45 s, extension at 72 °C for 1 min (35 cycles), and final extension at 72 °C for 10 min (1 cycle).

The PCR product was purified by using E.Z.N.A. Gel Extraction Kit (Omega bio-tech, USA) and was ligated into pMD19-T cloning vector (Takara Biotechnology, China), which was transformed into the *E. coli* strain (DH5α). The positive recombinant clones confirmed by restriction digestion were sequenced by Invitrogen Bio-tech (Shanghai, China), and then obtained results were analyzed by DNAssist software version 2.2. The HcES-15 gene was then cloned into *BamH* I/*EcoR* I restriction sites of expression plasmid pET32a (+) vector (Novagen, Madison, WI, USA). Then, the recombinant plasmid was sequenced again for confirmation of rHcES-15 gene insertion in the correct reading frame.

The recombinant plasmid pET32a (+)-HcES-15 was co-cultured in Luria-Bertini (LB) medium with ampicillin (100 µg/mL) for 3 h until optimal density of the culture reached at OD_600_, and expression of the fusion recombinant protein in *E. coli* BL21 cells (DE3) was induced by IPTG with 1 mM final concentration for 6 h at 37 °C. The fusion protein was purified from the supernatant of bacterial lysates using the His•Bind^®^ Resin Chromatography kit (Novagen) and dialyzed in phosphate buffered saline (PBS, pH 7.4) to remove imidazole. Endotoxins from the recombinant proteins were removed using ToxinEraser^TM^ Endotoxin Removal kit (GeneScript, Piscataway, NJ, USA). The purity and concentration of the purified rHcES-15 was resolved at 12% sodium dodecyl sulfate polyacrylamide gel electrophoresis (SDS–PAGE) and stained with Coomassie brilliant blue. The concentration of recombinant protein fraction was determined according to the Bradford procedure [53], using bovine serum albumin (BSA) as a standard and then stored at −20 °C for functional analysis.

### 4.6. Alignments and Phylogenetic Analysis

Local alignment search tools such as BLASTp and BLASTx were used for sequence similarity (http://www.blast.ncbi.nlm.nih.gov/Blast.cgi). ClustalX 1.83 program (http://www.clustal.org/) was used to align sequences of ES-15 proteins. The comparison between proteins from different parasites and phylogenetic tree based on the neighbor-joining method were analysed using the Molecular Evolutionary Genetics Analysis MEGA v6.0 software (Institute of Molecular Evolutionary Genetics, Penn State University, State College, PA, USA, http://www.megasoftware.net/) [54]. The protein sequence was used to predict N-terminal signal peptides within its structure (http://www.cbs.dtu-dk/services/SignalP/), as well as membrane protein prediction (http://www.cbs.dtu.dk/services-/TMHMM/) by using bioinformatics search tools.

### 4.7. Production of Antibodies

To generate polyclonal antibodies against rHcES-15, about 0.3 mg of rHcES-15 protein was mixed with Freund’s complete adjuvant 1:1 mixture and injected subcutaneously into SD rats at multiple places. After two weeks, rats received a booster dose of the same protein concentration with Freund’s incomplete adjuvant. Three booster doses were given to rats at 1-week intervals and then rats were anesthetized to collect blood containing specific anti-rHcES-15 antibodies in sera. Sera collected before protein injection was used as negative sera.

### 4.8. Immunoblot Analysis for the rHcES-15

The recombinant HcES-15 protein, after separation at 12% SDS-PAGE was transferred to polyvinylidene difluoride (PVDF) Membrane (Millipore, USA) for Western blot analysis as stated previously [55]. After blocking non-specific binding with 5% skim milk in Tris-buffered saline containing 0.1% Tween-20 (TBST), the membranes were then washed 3 times with TBST, and incubated with the primary antibodies (anti-rHcES-15) for 1 h at 37 °C (1:100 dilution in TBST). The membranes were then washed thrice and incubated with HRP-conjugated rabbit anti-rat IgG (Sigma, USA) for 1 h at 37 °C (diluted 1:3000 in TBST). Finally, the bound antibodies were detected using 3, 3-diaminobenzidine tetra hydrochloride (DAB) kit (Boster Biotechnology, Wuhan, China) according to the manufacturer’s instructions.

### 4.9. Binding of rHcES-15 to Goat PBMCs

The fresh isolated goat PBMCs were incubated with rHcES-15 protein or pET32a control protein (10 µg/mL-each) or PBS control for 1 h at 37 °C and protein binding to cells was determined by immunofluorescence assay (IFA) as previously described [30]. Briefly, the cells (1×10^5^/mL) were fixed with 4% paraformaldehyde on a poly-l-lysine-coated glass slide and blocked with 4% BSA in PBS solution for 30 min. After sequential incubation with rat anti-rHcES-15-IgG (1:100) for 2 h, the cells were washed with PBS and incubated with secondary antibody (1:500) coupled with Cy3 fluorescent dye (Beyotime, Jiangsu, China) for 1 h and for nuclear staining with 2-(4-amidinophenyl)-6-indole carbamidinedihydrochloride (DAPI, 1.5 μM; Sigma, MO, USA) for 6 min. The protein localization was visualized, after adding Anti-Fade Fluoromount solution (Beyotime Institute of Biotechnology, Nanjing, China) at 100× oil immersion objective lens on a laser scanning confocal microscope (L SM710, Zeiss, Jena, Germany). Digital images were captured using the Zeiss microscope software package ZEN 2012 (Zeiss, Jena, Germany).

### 4.10. Localization of HcES-15 in Adult H. contortus Worms

Freshly collected *H. contortus* adult worms were fixed in 4% formaldehyde–0.2% glutaraldehyde in PBS for 45 min and then dipped in TISSUE-TeK^®^ O.C.T. compound (SAKURA Finetek, Torrance, CA, USA). Immunohistochemical analysis was carried out to identify localization of HcES-15 in worm sections [55]. After being snap frozen in liquid nitrogen, worms were cut into cryostal sections of 10 μm thickness using cryotome (CM1950, Leica Biosystems, Nussloch, Germany). For immunohistochemical analysis, the sections were treated with 10% normal goat serum in PBS for 1 h to prevent non-specific binding, and then incubated with specific rat-anti-rHcES-15 serum (1:100 dilutions) or normal rat serum (control) for 2 h at 37 °C. After washing three times with PBS, the sections were incubated with secondary antibody coupled with Cy3, goat anti-rat IgG for 1 h at 37 °C. To stain corresponding nuclei within worm sections, DAPI (Beyotime Institute of Biotechnology, Nanjing, China) was used for 5 min and washed thrice with PBS. Finally, the specimens were immersed in Anti-Fade Mounting Medium (Beyotime Institute of Biotechnology, Nanjing, China) to prevent fading during microscopic examination.

### 4.11. Analysis of Cytokine Levels of PBMCs Treated with rHcES-15

Freshly isolated PBMCs (1 × 10^6^/mL) were re-suspended in complete medium (RPMI 1640) supplemented with 100 U/mL penicillin, 100 µg/mL streptomycin, 2 mM l-glutamine, and 10% fetal bovine serum (FBS). The cell viability was assessed by means of the trypan blue exclusion test before the incubation of PBMCs with rHcES-15. The cells were seeded into 24-well plates (1 mL/well) and treated with Concanavalin A (ConA: 10 µg/mL) alone or in the presence of different concentrations of rHcES-15 (10, 20, 40, and 80 µg/mL). The control groups were treated with equal volume of PBS or ConA with recombinant pET32a protein, for 72 h at 37 °C and 5% CO_2_. The production of IL-4, IL-10, IL-17, TGF-β1, and IFN-γ cytokines in supernatants were determined by using commercially available goat ELISA kits (Jiancheng Biotechnology, Nanjing, China) [56]. Three individual experiments were performed.

### 4.12. Cell Migration Assay

The cell migration assay was performed using a Millicell^®^ insert with 8.0 μm pores (Merck Millipore, Darmstadt, Hessen, Germany) according to the manufacturer’s instructions [57]. The freshly isolated PBMCs were incubated with different concentrations of rHcES-15 (10, 20, 40, and 80 µg/mL) and similarly, the control group was treated with an equal volume of PBS and pET32a protein (10 μg/mL) for 2 h at 37 °C and 5% CO_2_. The cells (200 µL) were seeded into the upper chamber and the lower chamber was filled with 1300 μL RPMI 1640 medium. Then, the cells migrated through the polycarbonate membrane into the lower chamber were determined by a Neubauer counting chamber. Each experiment was performed in triplicate.

### 4.13. Cell Proliferation Assay

About 100 μL of goat PBMCs suspension (1.5 × 10^6^ cells/mL) was activated with ConA (10 μg/mL) alone or containing serial concentrations of rHcES-15 (10, 20, 40, and 80 µg/mL). The control groups were treated with PBS or recombinant pET32a protein with ConA. The samples were poured in 96-well plate and were cultured at 37 °C and 5% CO_2_ for 72 h. Cell proliferation assay was performed by the addition of 10 μL of CCK-8 solution (Beyotime Institute of Biotechnology, Haimen, China) to each well, 4 h before harvesting, and the absorbance values were measured at 450 nm (OD_450_) using a microplate spectrophotometer (BioRad Laboratories, Hercules, CA, USA) [58]. Cells in the PBS group served as the control and the OD_450_ was set as 100%. The cells proliferation index was calculated by the formula: OD_450_ treatment/OD_450_ control.

### 4.14. Nitric Oxide Production Assay

The 100 μL of freshly harvested goat PBMCs (1 × 10^6^ cells/mL) was washed thrice in PBS and seeded in 96-well plates in DMEM (Dulbecco’s Modified Eagle Medium) culture medium. The cells were incubated with rHcES-15 (10, 20, 40, and 80 µg/mL) or without (PBS as control) and pET32a as recombinant empty protein. The cells were incubated for 24 h at 37 °C and intracellular nitric oxide production of PBMCs was determined by using the Griess assay, according to the instruction of Total Nitric Oxide Assay Kit (Beyotime Institute of Biotechnology, Shanghai, China). Absorbance of the colored solution in each well was measured at 540 nm (OD_540_) using a plate reader (BioRad Laboratories, Hercules, CA, USA). Absorbance values were converted to micromoles per liter (μmol/L) using a standard curve that was generated by addition of 0 to 80 μmol/L sodium nitrite to fresh culture media. Three individual experiments were performed.

### 4.15. Cell Apoptosis Assay

According to the manufacturer’s instructions of Annexin V-FITC kit (Miltenyi Biotec, Bergisch Gladbach, Nordrhein-Westfalen, Germany), apoptosis analysis was performed. PBMCs (1.5 × 10^6^ cells/mL) were cultured with control buffer (PBS), recombinant protein of pET32a, and with different protein concentrations of rHcES-15 in temperature and humidity-controlled conditions for 24 h. The cells were then washed twice with PBS (Ca^2+^/Mg^2+^-free, pH 7.4), re-suspended in binding buffer and the apoptotic assay was performed as per kit instructions and was checked on flow cytometer (BD Biosciences, San Jose, CA, USA). Results were analyzed using FlowJo 7.6 software (Tree Star, Ashland, OR, USA).

### 4.16. Cell Phagocytosis Activity

The phagocytic activity of freshly collected monocytes was measured as reported previously [57]. Briefly, cells were resuspended in 100 µL cold PBS after stimulated with rHcES-15 for 48 h. After that, cells were incubated with equal volume (1 mg/mL) of FITC-dextran (Sigma, St Louis, MO, USA) in RPMI 1640 at 4 °C and 37 °C for 1 h, and the reaction was stopped using cold PBS containing 2% FBS. Cells were washed three times and resuspended in PBS containing 2% paraformaldehyde. The FITC-dextran internalization of monocytes was analyzed by flow cytometry (BD Biosciences, San Jose, CA, USA), and results were analyzed using FlowJo 7.6 software (Tree Star, Ashland, OR, USA). The cells phagocytosis index was calculated by considering the statistical data of median fluorescence intensity (MFI) values in the control as 100%.

### 4.17. Statistical Analysis

Data are presented as mean ± SEM using the statistical package, GraphPad Premier 6.0 (GraphPad Prism, San Diego, CA, USA). The differences between groups were compared by one-way ANOVA, followed by a Tukey test and were considered statistically significant at *p* < 0.05.

## 5. Conclusions

In conclusion, our results demonstrated that rHcES-15 derived from the 15 kDa family of proteins, in interaction with host immune cells, elicited distinct immunomodulatory functions. The binding patterns of rHcES-15 played crucial roles in cytokines expression, cell proliferation, migration, NO production, apoptosis, and phagocytosis with goat PBMCs. These results do not only contribute to comprehend the functions of *H. contortus* low molecular weight antigen but might also help to elucidate the immune evasion mechanisms by parasites during host–parasite interactions. However, the actual biological pathways involved in cytokines-based cellular immunity during differential parasitic stage, level of infection, and host defense strategies should be included in future research.

## Figures and Tables

**Table 1:** Nucleotides and protein sequence similarity of the *H. contortus* ES-15 antigen (rHcES-15) by using bioinformatics search tools.

Sequence Type	Nucleotides/Proteins Description	Identity (%)	Accession Number (GenBank)
Nucleic Acid	*H. contortus* 15 kDa excretory/secretory protein	96	AY821552.1
	*H. contortus* 15 kDa excretory/secretory protein	95	U64792.1
	*H. contortus* isolate Bareilly p15	95	JF826244.1
	*Haemonchus placei* genome assembly	96	LM596575.1
		96	LM589008.1
		94	LM588224.1
		95	LM585135.1
		95	LM591864.1
		91	LM587365.1
		86	LM594683.1
Amino Acid	15 kDa excretory/secretory protein (*H. contortus*)	100	AAV84000.1
	p15 (*H. contortus*)	97	CDJ85218.1
	15 kDa excretory/secretory protein (*H. contortus*)	92	CDJ84355.1
	p15 (*H. contortus*)	92	CDJ85217.1
	15 kDa excretory/secretory protein (*H. contortus*)	89	O18518.1
	p15 (*H. contortus*)	88	AEG76953.1
	15 kDa excretory/secretory protein (*H. contortus*)	81	CDJ84626.1
	p15 (*H. contortus*)	83	CDJ84625.1
	15 kDa excretory/secretory protein (*H. contortus*)	85	CDJ84539.1
	p15 (*H. contortus*)	95	CDJ84538.1
	p15 (*H. contortus*)	88	CDJ85219.1
	30 kDa antigenic glycoprotein (*T. colubriformis*)	38	O97391.1
	Unnamed protein product (*H. contortus*)	25	CDJ84854.1
	Unnamed protein product (*H. contortus*)	30	CDJ81999.1

**Table 2:** Specific set of primers used for PCR amplification.

Name	Sequence (5′ – 3′)	GenBank Accession Number	Restriction Sites
HcES-15 (Forward)	AAA*GGATCC*ATGTTCTTCGC TTTTGC	AY821552.1	*BamH I*
HcES-15 (Reverse)	CTG*GAATTC*TCAGTTGGGGGTATTGT		*EcoR I*

## Supplementary Materials

Supportive data and materials regarding this study are available online at https://www.mdpi.com/2076-0817/9/3/162/s1, Figure S1: PCR amplification and recombinant plasmids confirmation for HcES-15 gene, Figure S2: Multiple sequence alignment of amino acid for HcES-15, Figure S3: N-terminal signal peptide prediction, Figure S4: Membrane protein prediction using TMHMM Server v.2.0.
