# Supplementary material for: Characterization of Haemonchus contortus Excretory/Secretory Antigen (ES-15) and Its Modulatory Functions on Goat Immune Cells In Vitro
Source: Pathogens. 2020 Feb 27;9(3):162. doi: 10.3390/pathogens9030162 (PMC7157690; doi:10.3390/pathogens9030162)
Supplement: Supplementary file 1 [file pathogens-09-00162-s001.pdf]

Supplementary Materials

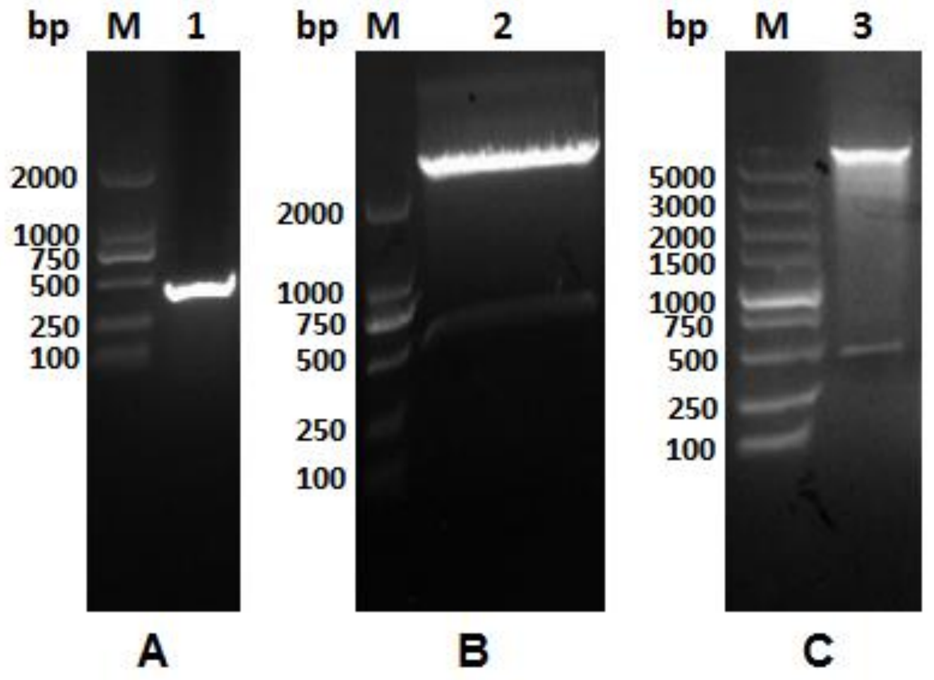

Figure S1. PCR amplification and recombinant plasmids confirmation for HcES-15 gene. M: Molecular weight marker. PCR product for HcES-15 **(A)**, cloned into pMD-19T vector **(B)** and pET32a vector **(C)**, confirmed by restriction enzyme digestion with *BamH* I and *EcoR* I.

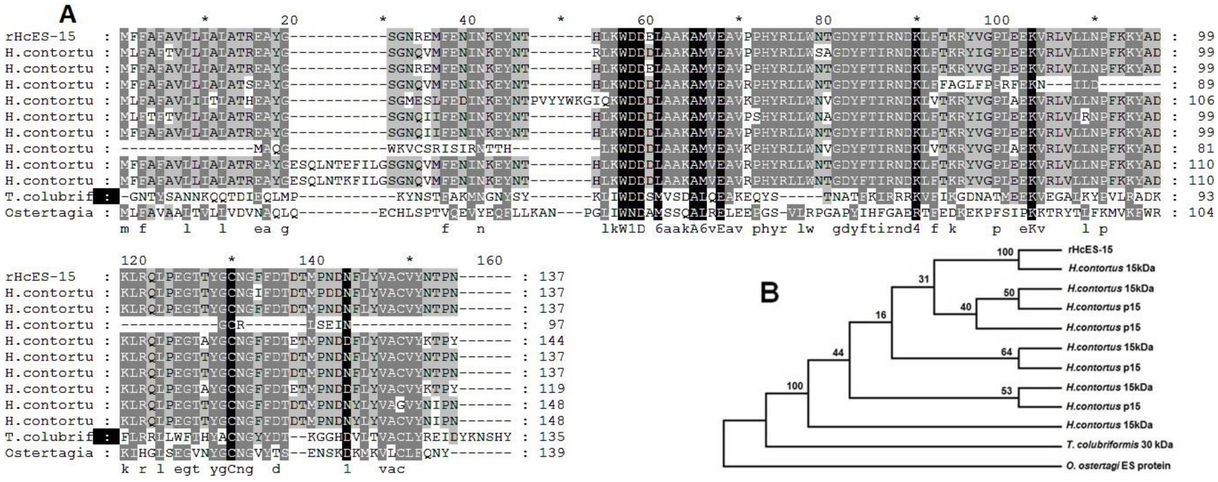

Figure S2. Multiple sequence alignment of amino acid for HcES-15. (A): The protein sequence of HcES-15 aligned with *H. contortus* ES antigens reported in the NCBI database, *H. contortus* 15 kDa excretory/secretory protein AAV84000.1 (100%), CDJ84355.1 (92%), O18518.1 (89%), CDJ84539.1 (85%) and CDJ84626.1 (81%), *H. contortus* p15 CDJ85218.1 (97%), CDJ85217.1 (92%), AEG76953.1 (88%) and CDJ84625.1 (83%), *O. ostertagi* putative L3 ES protein CAH23216.1 (26%), *T. colubriformis* 30 kDa antigenic glycoprotein O97391.1 (38%). (B): Phylogenetic relationship of deduced amino acid sequences of HcES-15 with other known sequences. The values on branches indicate bootstraps for 1000 replicates.

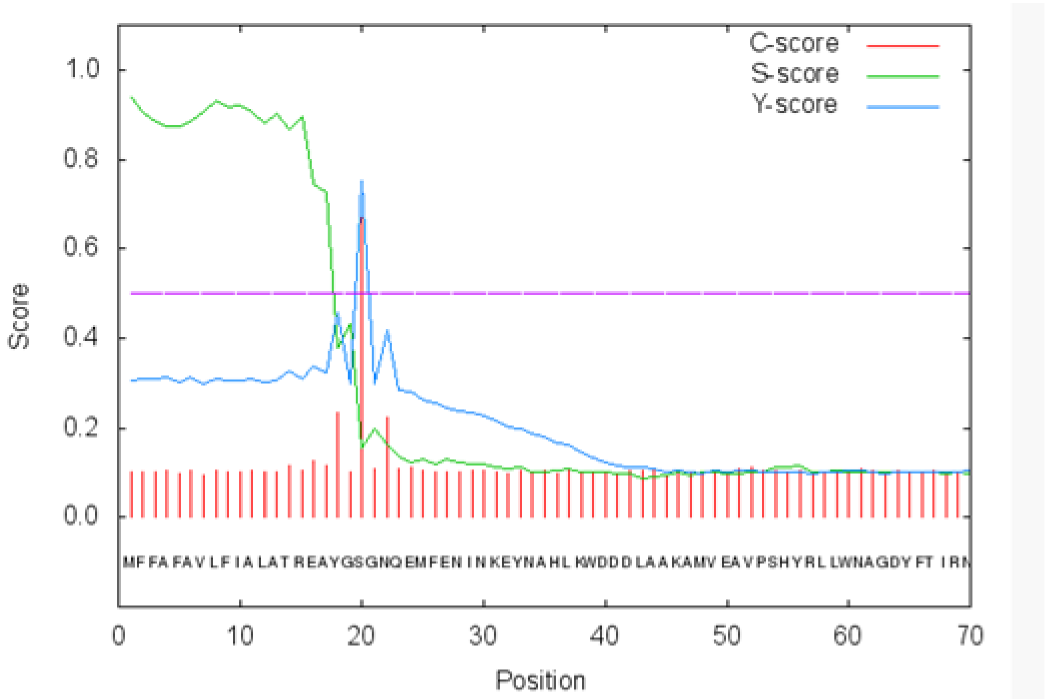

Figure S3. N-terminal signal peptide prediction. The amino acid sequences of HcES-15 (NCBI accession numbers: AY821552.1) was used to predict N-terminal signal peptides by SignalP 4.1 Server (http://www.cbs.dtu.dk/services/SignalP/).

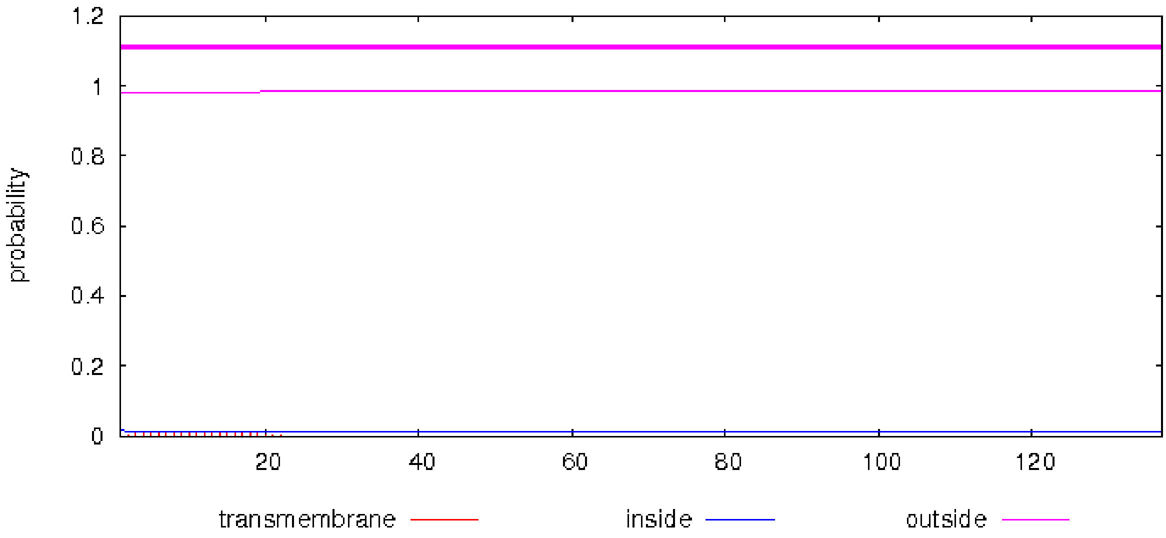

Figure 4. Membrane protein prediction using TMHMM Server v.2.0. The amino acid sequences of HcES-15 (NCBI accession numbers: AY821552.1) was analyzed to predict transmembrane structures using TMHMM Server v.2.0. There were no transmembrane domains was predicted in this protein structure. http://www.cbs.dtu.dk/services/TMHMM/.
